# First UK use of bacteriophage therapy with DAIR for chronic *Staphylococcus aureus* prosthetic joint infection

**DOI:** 10.1128/asmcr.00082-26

**Published:** 2026-07-06

**Authors:** A. Scobie, T. Ferry, D. Roulston, S. Subbarao, T. Thai, G. Trafford, A. Cudmore, J. Miles, K. Crick, S. H. Chin, R. Lester, S. Warren, N. Karunaharan, P.-J. Ceyssens, M. Merabishvili, S. Djebara, J.-P. Pirnay, T. Azamgarhi

**Affiliations:** 1 ESCMID Study Group for Non-Traditional Antibacterial Therapy (ESGNTA), Basel, Switzerland; 2 Department of Infection, Royal Free London NHS Foundation Trust 4965, London, United Kingdom; 3 Royal National Orthopaedic Hospital NHS Trust 7597, London, United Kingdom; 4 Division of Infection and Immunity, University College London 4919, London, United Kingdom; 5 Infectious Diseases, Hôpital de la Croix-Rousse, Hospices Civils de Lyon, Lyon, France; 6 Centre de référence pour la prise en charge des infections ostéoarticulaires complexes, CRIOAc Lyon, Hospices Civils de Lyon, Lyon, France; 7 Claude Bernard Lyon 1 University, Lyon, France; 8 Division of Medicine, University College London, London, United Kingdom; 9 Centre for Clinical Microbiology, University College London, United Kingdom; 10 Royal Devon University Healthcare NHS Foundation Trust, Exeter, United Kingdom; 11 Antimicrobial Resistance and Healthcare Associated Infections Division (AMR & HCAI), UKHSA, London, United Kingdom; 12 Bacterial Diseases Unit, Sciensano, Brussels, Belgium; 13 Laboratory for Molecular and Cellular Technology, Queen Astrid Military Hospital, Brussels, Belgium; Vanderbilt University Medical Center, Nashville, Tennessee, USA

**Keywords:** bacteriophage or phage therapy, prosthetic joint infection, *Staphylococcus aureus*, salvage therapy, antimicrobial resistance

## Abstract

**Background:**

Chronic *Staphylococcus aureus* prosthetic joint infection (PJI) remains difficult to manage when surgical revision and long-term antibiotics are not feasible. Bacteriophage therapy is emerging as a potential adjunct, though experience in orthopedic infections, particularly in the United Kingdom, is limited.

**Case Summary:**

We report the first UK case of intra-articular bacteriophage therapy administered alongside debridement, antibiotics, and implant retention (DAIR) for chronic methicillin-sensitive *Staphylococcus aureus* knee PJI. An 81-year-old man with multiple comorbidities developed persistent infection following revision knee arthroplasty, with recurrent sinus formation despite multiple surgical washouts and prolonged suppressive antibiotics. Major revision surgery and amputation were not viable options. Following a multidisciplinary review through the UK Clinical Phage Network, targeted phage therapy was pursued as salvage treatment. Phage susceptibility testing identified an active lytic phage (ISP). The patient underwent open DAIR with intra-articular phage administration, adjunctive local antibiotics, short-course intravenous antimicrobials, and subsequent oral suppressive therapy. Two further intra-articular phage doses were administered postoperatively. Initial sinus closure occurred, but recurrence developed within 4 weeks. At 18 months, symptoms were partially improved with better mobility and reduced inflammation, though one sinus tract persisted. No significant adverse effects were observed. Whole-genome sequencing of pretreatment isolates demonstrated a predominantly ST5 *S. aureus genotype* with conserved biofilm-associated virulence genes and limited antimicrobial resistance in addition to clonal diversification consistent with chronic biofilm infection.

**Conclusion:**

This case demonstrates the feasibility and safety of intra-articular phage therapy during DAIR in a UK setting but highlights biological, logistical, and pharmacological factors that may limit efficacy in advanced chronic PJI.

## INTRODUCTION

Prosthetic joint infections (PJIs) caused by *Staphylococcus aureus* remain among the most challenging complications in orthopedic surgery. Definitive treatment requires eradication of biofilms, typically through debridement and either implant removal, exchange, or amputation. In patients with significant comorbidities, however, definitive surgical treatment such as implant exchange may be contraindicated or declined.

Phage therapy (PT)—the administration of bacteriophage viruses that infect and kill target bacteria—has re-emerged as a potential treatment adjunct. We report the first UK case of intra-articular PT administration alongside debridement, antibiotics, and implant retention (DAIR) for chronic *Staphylococcus aureus* PJI and evaluate the factors contributing to a partial response.

## CASE PRESENTATION

An 81-year-old male with a background of ischemic heart disease with chronic left ventricular thrombus, chronic kidney disease stage 3, asthma, abdominal hernia repair, and bilateral knee replacements underwent a left total knee replacement (TKR) in 1989 and a further revision to a Mega-C distal femoral endoprosthesis in 2019 due to femoral loosening. Twelve months later, he developed chronic *methicillin-sensitive Staphylococcus aureus* (MSSA) PJI.

Despite multiple surgical interventions—including DAIR in June 2021, arthroscopic washout in 2022, and posterior abscess drainage in 2023—the infection persisted, with recurrent sinus formation and repeated isolation of MSSA. The organism remained susceptible to several agents, including doxycycline and flucloxacillin, but management was complicated by multiple severe antibiotic allergies and intolerances (penicillin, clindamycin, dalbavancin, and rifampicin). Long-term suppressive antibiotic therapy failed to control symptoms. Due to his comorbidities, major revision surgery was deemed unsuitable, and he declined amputation.

**Fig 1:**
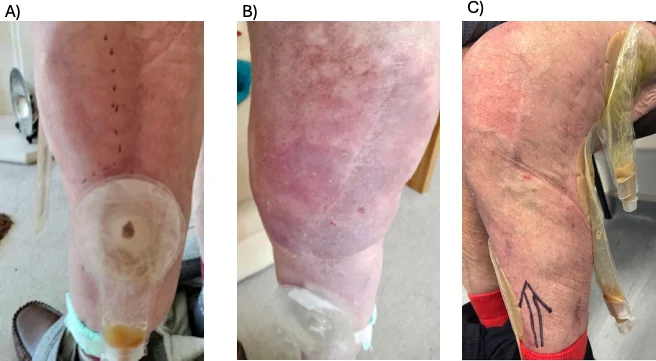
(**A and B**) Photographs illustrating anterior and posterior sinuses with associated calf abscess (June 2024). (**C**) Photograph demonstrating development of a third sinus (October 2024).

**Fig 2:**
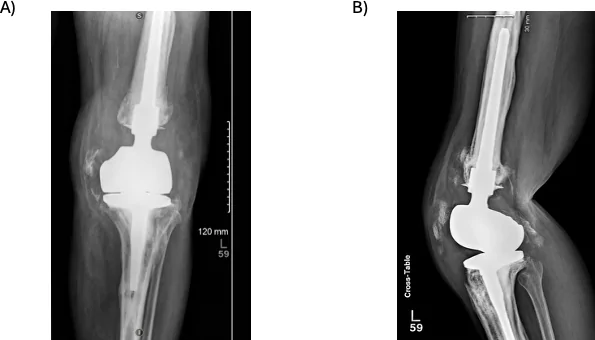
Preoperative radiographs. (**A**) Anteroposterior view demonstrating a well-aligned cemented hinged distal femoral endoprosthesis with no evidence of loosening, subsidence, or periprosthetic fracture. (**B**) Lateral view confirming stable positioning of the femoral and tibial components, intact cement mantle, and absence of implant migration or mechanical failure.

By September 2023, he had two actively discharging sinuses (Fig. 1) with a raised C-reactive protein (CRP) of 100 mg/L. Imaging (Fig. 2) demonstrated a stable prosthesis without radiographic loosening.

**Fig 3:**
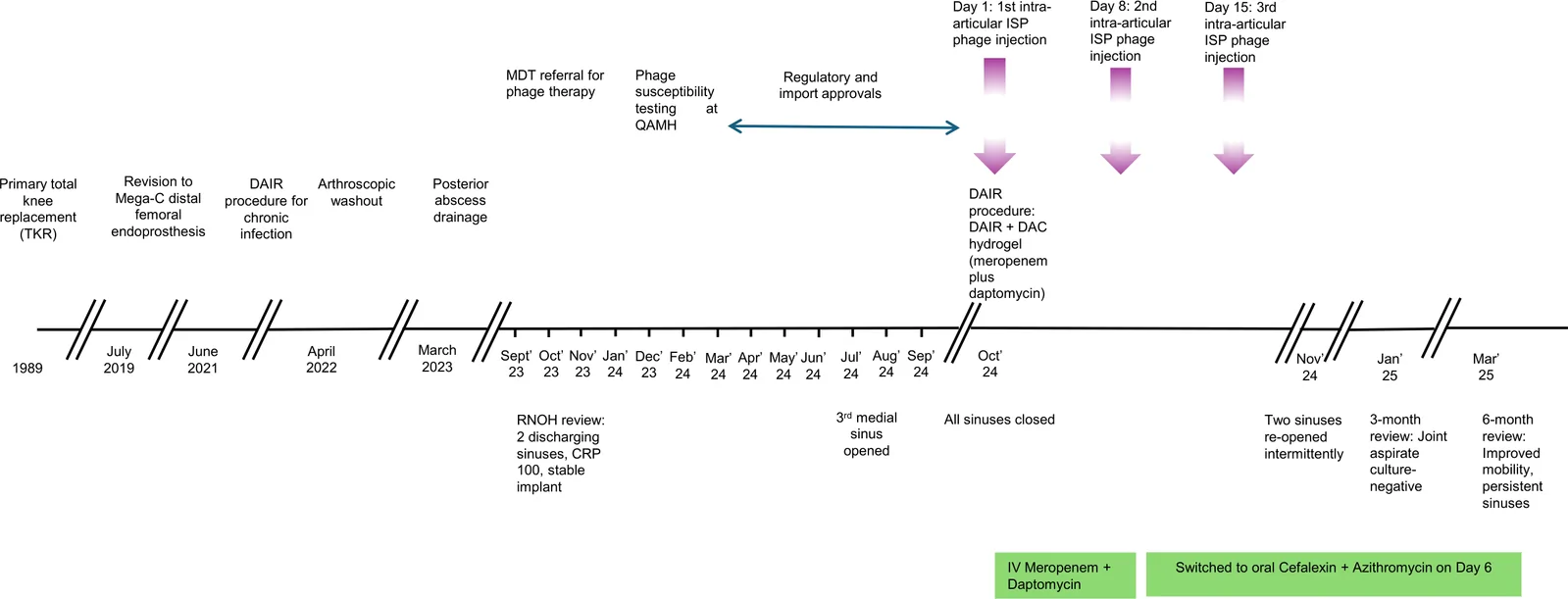
Chronological timeline of clinical events, antimicrobial treatment, and phage therapy. This figure illustrates the full clinical course from the patient’s original knee replacement in 1989 through to the 18-month follow-up after phage therapy in 2025. Key surgical interventions and infectious complications (including sinus formation and recurrence) are shown along the baseline. Phage therapy-related time points are marked with vertical purple arrows, including the three intra-articular ISP phage injections administered on Days 1, 8, and 15 following DAIR. The prolonged regulatory and import approval process is annotated above the timeline. Antimicrobial therapy is summarized in the green box. Breaks in the timeline denote periods without major intervention. Abbreviations: TKR, total knee replacement; DAIR, debridement, antibiotics, and implant retention; CRP, C-reactive protein; IA, intra-articular; QAMH, Queen Astrid Military Hospital.

The case was reviewed by the UK Clinical Phage Network, an external multidisciplinary group including infectious diseases, microbiology, orthopedics, and international phage experts (Fig. 3). Phage therapy was recommended alongside DAIR with the aim of symptom control and avoiding amputation.

**Fig 4:**
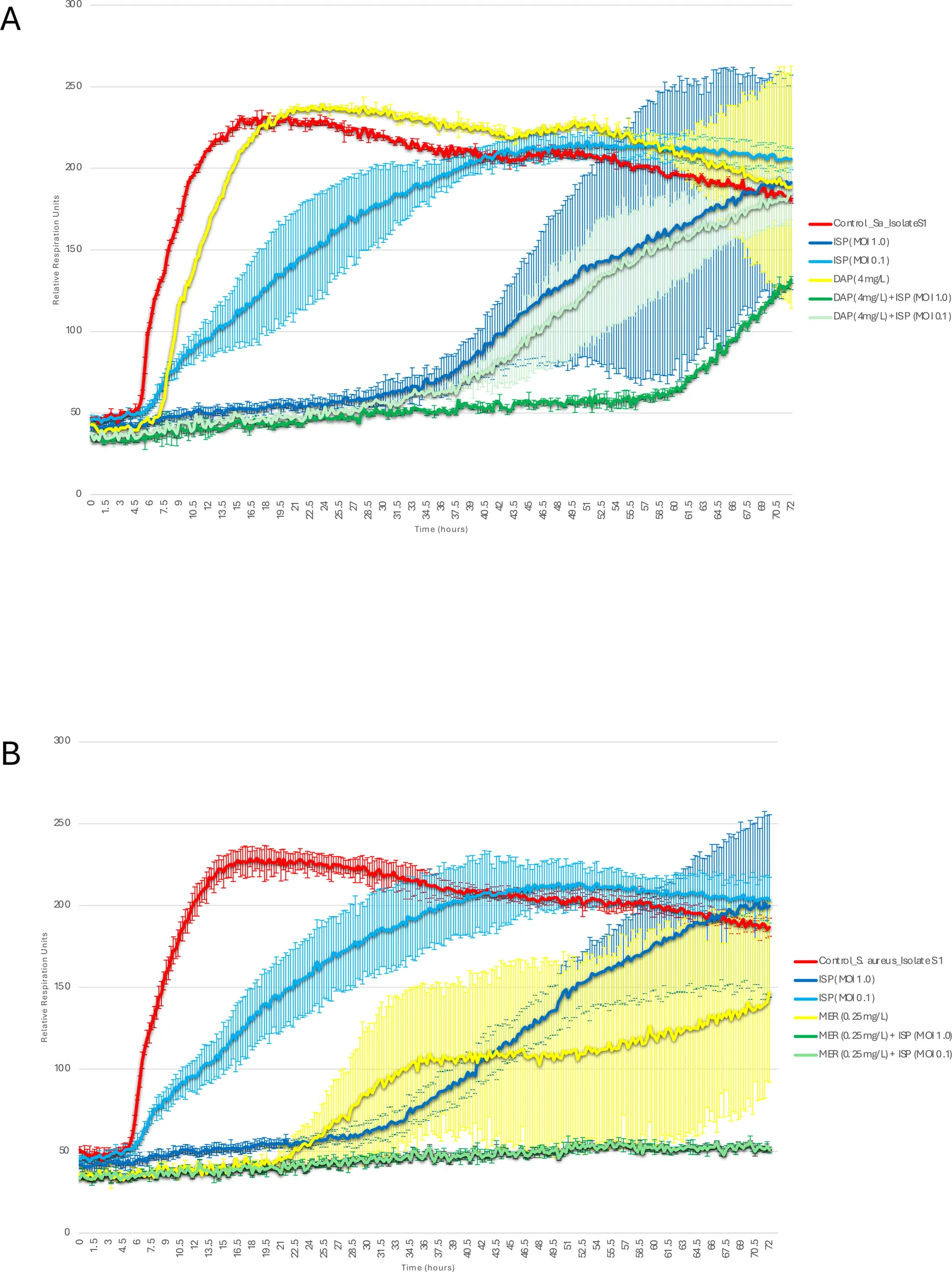
Phage-antibiotic interaction tested with the OmniLog system for daptomycin (**A**) and meropenem (**B**). Growth curves show bacterial metabolic activity in liquid broth in the presence of phage alone, antibiotic alone, and the combination across a range of concentrations. Each test was performed in technical triplicate, with curves generated using the median values of these replicates; error bars represent the absolute median deviations. A downward shift of the combination curve relative to either agent alone indicates synergistic killing. MOI = multiplicity of infection (the ratio of infectious virions to bacterial cells at the start of the assay). These data informed antibiotic selection for both intraoperative and postoperative phases of treatment.

Bacterial isolates were sent for phage susceptibility testing and phage-antibiotic interaction testing at Queen Astrid Military Hospital, Belgium. Initial screening for phage activity was performed using standard plaque assays to identify lytic phages active against the patient isolate. Two active lytic phages (ISP and Phage 25) targeting the *S. aureus* strain were identified. Phage–antibiotic interaction testing was subsequently performed using the OmniLog system, a liquid-based phenotypic assay that measures bacterial growth kinetics in the presence of phage and antibiotics across multiple multiplicities of infection (MOI) and antimicrobial concentrations. Strong synergistic activity was observed between ISP and doxycycline, linezolid, daptomycin, and meropenem across several MOI ranges. Representative OmniLog growth curves demonstrating synergy between ISP and daptomycin and meropenem are shown in Fig. 4. Neutral or additive effects were observed with ciprofloxacin, gentamicin, and cefotaxime, while antagonistic or neutral effects were seen with co-trimoxazole and amikacin. Due to regulatory delays, Phage 25 expired prior to treatment, and only ISP was available for clinical use (1).

**Fig 5:**
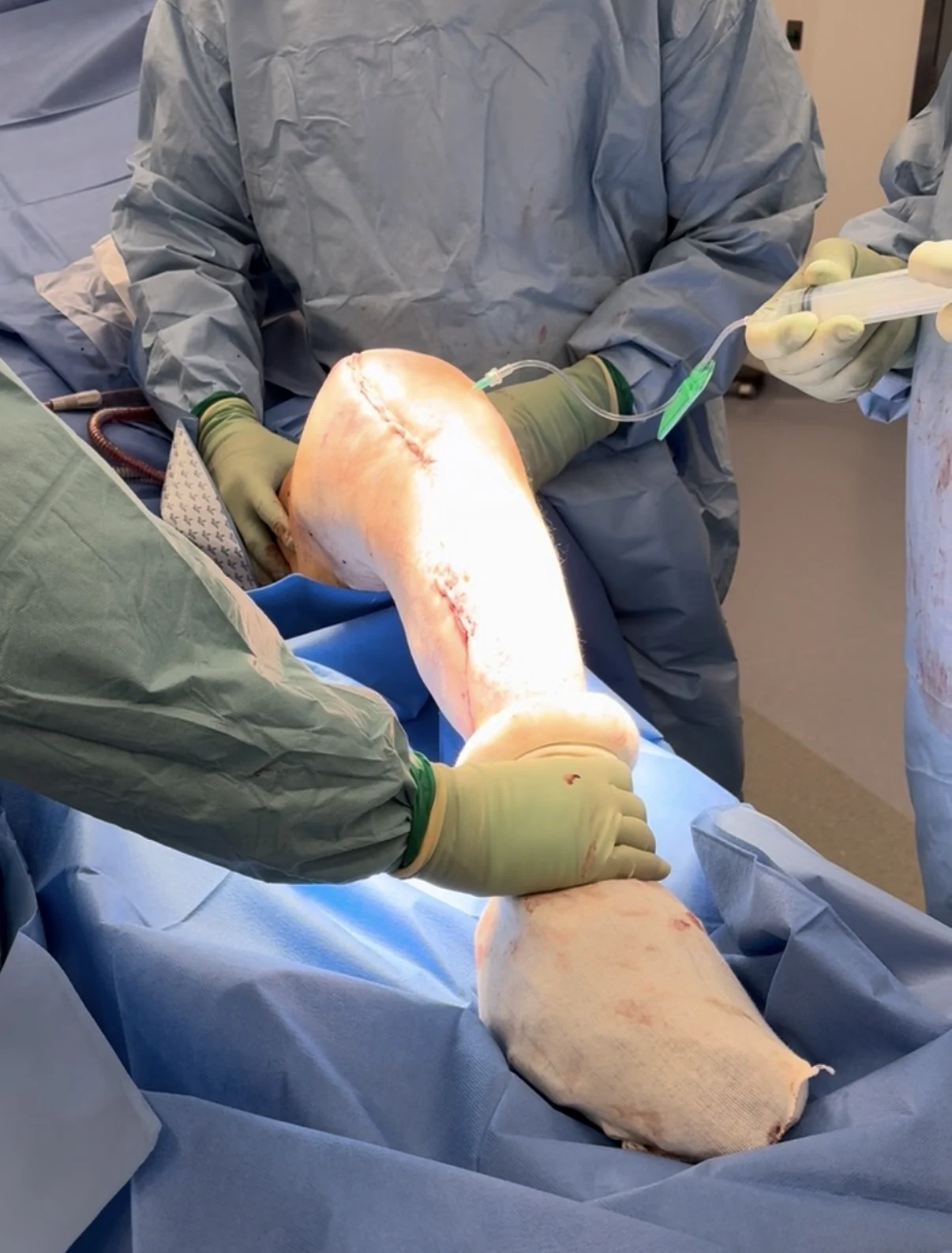
Intra-articular administration of ISP phage following DAIR and wound closure.

In October 2024, following approvals, phage therapy was administered intra-articularly following the published “PhagoDAIR” procedure (2) (Fig. 5). Surgery revealed frank pus and extensive synovitis. Following aggressive debridement and sinus excision, local antibiotics (daptomycin and meropenem) were delivered via a degradable hydrogel (Defensive Antibacterial Coating, DAC) around the implant. After wound closure, ISP phage (10⁹ PFU/mL diluted in 30 mL 0.9% NaCl) was administered via intra-articular injection. The patient received 5 days of meropenem 1 g t.d.s and daptomycin (10 mg/kg) o.d. before conversion to long-term oral suppression with cefalexin 1 g t.d.s and azithromycin 500 mg o.d. This regimen was selected due to multiple antibiotic allergies and intolerances that limited alternative options, alongside phage–antibiotic interaction testing that demonstrated favorable interactions and supported the use of a tolerable long-term oral regimen. Two additional intra-articular phage injections were administered under ultrasound guidance on days 8 and 15 with the aim of maintaining local phage exposure during the early postoperative period. *Staphylococcus aureus* was isolated in 5/7 intraoperative culture samples. Colonial variation was noted with differing antibiotic susceptibility profiles. All isolates were susceptible to vancomycin, flucloxacillin, clarithromycin, and daptomycin and resistant to rifampicin. Monitoring was performed in accordance with international published protocols (3–5). Apart from a transient febrile episode 48 h postoperatively, no adverse effects were observed.

All sinuses closed within 2 weeks of therapy; however, by week 4, two sinuses reopened with intermittent discharge. Joint aspiration was performed at 3 months while the patient remained on suppressive antibiotics. This was undertaken in part to attempt re-isolation of the organism to assess for the potential development of phage resistance; however, the aspirate was culture-negative, and therefore repeat susceptibility testing could not be performed. A synovial fluid cell count had also been requested but could not be performed as the sample clotted during laboratory processing.

A CT scan at 6 months demonstrated osteomyelitis and lucency around the tibial component with a large effusion. Although preoperative CT imaging was unavailable for comparison, these findings were consistent with ongoing chronic infection. At 6 months, the patient reported improved mobility, reduced swelling, and a subjective 70% improvement in symptoms, though persistent sinus tracts remained. At the 18-month follow-up, the patient remained clinically stable on suppressive antibiotic therapy with cefalexin alone and only one persisting sinus. Symptoms remained improved compared with baseline, with continued mobility and no progression of local disease. Importantly, the patient avoided further hospital admissions, intravenous antibiotics, and amputation.

### Genomic analysis

*Post hoc* whole-genome sequencing was performed on 19 *S. aureus* isolates to characterize the bacterial population and identify intrinsic prophage elements that might influence susceptibility to the administered phage. The analysis was not intended to detect integration of the therapeutic phage itself but rather to assess the presence of endogenous phage sequences within the bacterial genome. Isolates included the original screening isolates (S1 and S2) and multiple colonial variants recovered from intraoperative cultures.

The majority were sequence type (ST) 5, a lineage commonly associated with healthcare-associated infections and biofilm formation. Table 1 summarizes the sequence type, efficiency of plating, and phage activity across isolates recovered before and during surgery. One isolate was ST434-like and carried both lukF-PV and lukS-PV, consistent with PVL positivity, though it remained closely related to the dominant population (Table 1).

**TABLE 1:** Phenotypic and phage susceptibility characteristics of *Staphylococcus aureus* isolates obtained before and during debridement, antibiotics, and implant retention (DAIR) for chronic prosthetic joint infection^*b*^

Sampling time point	Sequence type	Isolate ID	Efficiency of plating (EOP)^*a*^
Preoperative	ST5	S1	0.1
Preoperative	ST5	S2	0.1
Intraoperative	ST5	A1-CV1	0.1
Intraoperative	ST5	A1-CV2	0.01–0.1
Intraoperative	ST5	B1-CV1	0.2
Intraoperative	ST5	B1-CV2	0.07
Intraoperative	ST5	C2-CV1	0.01–0.1
Intraoperative	ST5	C2-CV2	0.01–0.1
Intraoperative	ST5	C3-CV1	0.01–0.1
Intraoperative	ST434	C3-CV2	0.03
Intraoperative	ST5	D1-CV2	0.03
Intraoperative	ST5	E1-CV1	0.03
Intraoperative	ST5	E2-CV1	0.5
Intraoperative	ST5	E3-CV1	0.08
Intraoperative	ST5	E3-CV2	0.07
Intraoperative	ST5	E3-CV3	0.01
Intraoperative	ST5	F1-CV1	0.04
Intraoperative	ST5	F2-CV1	0.2
Intraoperative	ST5	F3-CV1	0.01–0.1

^*a*^ Efficiency of plating (EOP) expressed relative to the propagating host strain.

^*b*^ Sequence types are shown alongside EOP results for all isolates recovered preoperatively and intraoperatively.

All isolates carried a conserved set of biofilm-associated genes, including the icaADBC operon and icaR, along with adhesins (clfA, sdrC, and sdrE) and the cap8 capsule locus. Type VII secretion system genes were also universally present, supporting a phenotype associated with persistence and immune evasion. Antimicrobial resistance gene carriage was concordant with phenotypic susceptibility testing. Genes associated with aminoglycoside, tetracycline, fluoroquinolone, phenicol, and fosfomycin resistance were detected.

**Fig 6:**
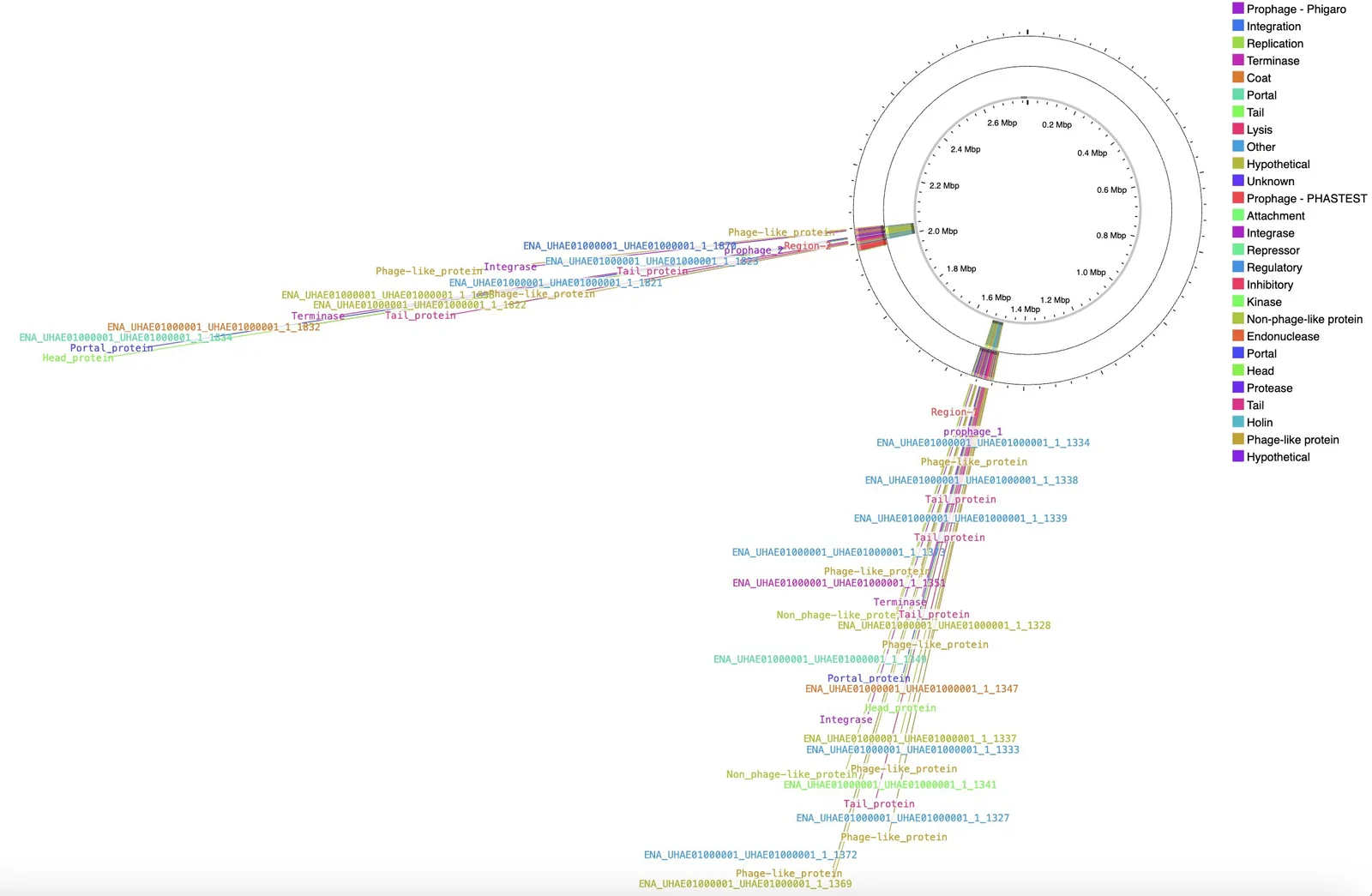
Annotated prophage regions in *S. aureus* ST5 (Isolate 1). Circular genome map showing two prophage regions identified via *post hoc* whole-genome sequencing. Genes are color-coded by predicted function, including integrases (teal), tail proteins (green), terminases (purple), coat proteins (red), and hypothetical proteins (orange). Region 1 aligned with Siphoviridae phages of the *Peeveelvirus* genus. Region 2 did not yield a definitive match on BLAST analysis. Prophage regions were conserved across all ST5 isolates and may represent endogenous phage elements within the host genome.

Prophage analysis identified two conserved phage regions across all ST5 isolates. One region showed alignment with phages of the *Peeveelvirus* genus (Class Caudoviricetes, Subfamily Bronfenbrennervirinae). The genomic organization of these prophage regions is illustrated in Fig. 6. These findings confirm the presence of clonal diversity and virulence determinants consistent with chronic biofilm-associated infection.

## DISCUSSION

This report describes the first documented UK case of bacteriophage therapy administered in conjunction with DAIR for chronic *Staphylococcus aureus* knee PJI. While several international case series describe favorable outcomes with phage therapy, its use in PJI remains uncommon and has often been reported without simultaneous surgical intervention (2, 6, 7). Recent reviews have also summarized the growing clinical experience with phage therapy in prosthetic joint infection and other orthopedic infections, highlighting both promising early outcomes and the need for further controlled studies (8, 9). Equally important to reporting successful cases is the description of partial or suboptimal responses as these provide critical insights into patient selection, disease stage, and treatment parameters that may influence outcomes (10, 11). This case adds novel clinical experience from a UK setting and highlights both the practical feasibility of phage therapy delivery and the biological and logistical limitations encountered in advanced chronic infection.

This case demonstrates that targeted phage therapy can be safely integrated into surgical management for PJI when conventional options are exhausted. Intraarticular phage administration was well tolerated, with no serious adverse events, and resulted in early sinus closure and sustained symptomatic improvement. However, infection eradication was not achieved, underscoring the challenges of treating long-standing biofilm-associated infection.

The precise reason for the limited clinical response in this case remains uncertain. Neither phenotypic nor genomic evidence of resistance to the administered phage was identified in the isolates available for testing. However, repeat susceptibility testing following therapy was not possible because the organism could not be re-isolated from the follow-up joint aspirate, and therefore the development of phage resistance during treatment could not be formally assessed. The partial response likely reflects a combination of biological, pharmacological, and logistical factors, which are discussed below.

Several factors likely contributed to the partial response in this case. Most notably, significant delays occurred between identification of phage susceptibility and treatment delivery due to regulatory approvals, logistical constraints, and the absence of domestic phage manufacturing or testing capacity. Although the phage was produced under a Belgian magistral framework with quality safeguards, uncertainty regarding equivalence to UK “GMP-like” standards contributed to prolonged approval timelines. These delays had practical consequences. During this period, local disease progression likely increased bacterial burden and biofilm maturity, reducing the likelihood of eradication. Delays also resulted in the loss of one phage (Phage 25) from the intended cocktail, necessitating monophage therapy. Increasing evidence supports the use of phage cocktails in chronic PJI to reduce resistance development and mitigate the effects of phage-neutralizing antibodies (12–16), which may have further limited treatment efficacy.

Additional procedural factors may also have played a role. To preserve phage viability, standard intraoperative measures such as antiseptic irrigation with chlorhexidine and negative pressure wound therapy were avoided. While necessary for phage delivery and local exposure, these deviations may have compromised overall infection control. Furthermore, limited data exist on intraarticular phage pharmacokinetics, particularly in the presence of draining sinuses. Persistent sinus tracts, ongoing drainage and peri-prosthetic dead space may reduce the residence time of intra-articularly administered phage and limit effective bacterial exposure within the joint space. These anatomical factors may reduce local phage concentrations and represent a potential barrier to treatment success. In such situations, alternative delivery strategies, including higher local titers, repeated dosing, or combined systemic administration, may warrant consideration, although clinical data remain limited. Potential interactions between phages and locally applied antibiotic carriers remain poorly understood and represent an important area for further investigation. The dosing strategy used in this case was informed by the published PhagoDAIR procedure together with guidance from international centers experienced in clinical phage therapy. Intraarticular administration was selected in order to maximize local phage exposure at the site of infection following surgical debridement. Intravenous phage therapy was not pursued due to regulatory uncertainty regarding systemic administration of a non-GMP phage preparation within the United Kingdom at the time of treatment. As regulatory frameworks evolve and manufacturing capacity develops, combined systemic and local delivery strategies may become more feasible and may offer pharmacokinetic advantages in complex infections.

The dosing strategy was also conservative. Three intraarticular phage injections were administered without concurrent intravenous phage therapy or continuous local delivery via a drain, as described in some successful reports (2, 6). This approach reflected pragmatic constraints within an NHS inpatient setting. Reconstitution in saline resulted in lower phage titers (3.3 × 10^8^ PFU/mL) than those reported in some effective PJI cases (2, 8, 16, 17). Optimal dosing, frequency, and routes of administration for phage therapy in orthopedic infection remain undefined, and higher phage titers or combined systemic and local exposures may be required in advanced disease. *Post hoc* genomic analysis provided further insights into biological barriers to treatment success. The infection was dominated by *S. aureus* ST5, with minor genomic variation consistent with within-host evolution in a chronic biofilm environment. All isolates harbored conserved biofilm-associated genes and type VII secretion systems, supporting persistence and immune evasion. Limited antimicrobial resistance gene carriage was observed, aligning with phenotypic susceptibility testing. Conserved prophage elements were present, although no inducible phage elements likely to interfere with therapy were identified. Despite marked colonial variation within the *S. aureus* isolates, phenotypic and genotypic resistance to ISP phage was not detected. The role of phage-neutralizing antibodies also remains uncertain as testing was not possible due to loss of samples in transit.

This case reinforces the value of phage-antibiotic interaction testing to optimize therapy. In this case, synergy was demonstrated between ISP and several antibiotics, which informed both systemic and local antimicrobial choices, while antagonism with co-trimoxazole and amikacin constrained options despite conventional susceptibility results. These findings support an individualized, precision-medicine approach to phage therapy.

Local approval for phage therapy was strengthened by an external multidisciplinary team (MDT) review coordinated through the UK Clinical Phage Network. Given the limited national expertise in this emerging field, this external input was critical in reassuring local governance structures and enabling access. As phage therapy expands, dedicated national centers are required to streamline access, reduce delays, and standardize delivery and data collection, as seen in other countries (4, 7, 10, 15).

### Conclusion

In conclusion, this case demonstrates that intraarticular phage therapy during DAIR is feasible and safe in a UK setting but may offer limited benefit in advanced chronic PJI. While symptom improvement and limb preservation were achieved, earlier intervention, phage cocktails, optimized dosing strategies, and improved infrastructure may be critical for better outcomes. Reporting such cases is essential to define patient selection, identify potential predictors of failure, and guide future clinical trials.

## References

[1] Pirnay J-P, Verbeken G, Ceyssens P-J, Huys I, De Vos D, Ameloot C, Fauconnier A. 2018. The magistral phage. Viruses 10:64. doi:10.3390/v10020064 29415431 PMC5850371

[2] Ferry T, Kolenda C, Batailler C, Gustave C-A, Lustig S, Malatray M, Fevre C, Josse J, Petitjean C, Chidiac C, et al.. 2020. Phage therapy as adjuvant to conservative surgery and antibiotics to salvage patients with relapsing *S. aureus* prosthetic knee infection. Front Med 7:570572. doi:10.3389/fmed.2020.570572 PMC7701306 33304911

[3] Khatami A, Foley DA, Warner MS, Barnes EH, Peleg AY, Li J, Stick S, Burke N, Lin RCY, Warning J, et al.. 2022. Standardised treatment and monitoring protocol to assess safety and tolerability of bacteriophage therapy for adult and paediatric patients (STAMP study): protocol for an open-label, single-arm trial. BMJ Open 12:e065401. doi:10.1136/bmjopen-2022-065401 PMC9743374 36600337

[4] Onallah H, Yerushalmy O, Braunstein R, Alkalay-Oren S, Rimon A, Gelman D, Coppenhagen-Glazer S, Hazan R, Nir-Paz R. 2024. Protocol for phage matching, treatment, and monitoring for compassionate bacteriophage use in non-resolving infections. STAR Protoc 5:102949. doi:10.1016/j.xpro.2024.102949 38691464 PMC11070627

[5] Onsea J, Uyttebroek S, Chen B, Wagemans J, Lood C, Van Gerven L, Spriet I, Devolder D, Debaveye Y, Depypere M, et al.. 2021. Bacteriophage therapy for difficult-to-treat infections: the implementation of a multidisciplinary phage task force (The PHAGEFORCE study protocol). Viruses 13:1543. doi:10.3390/v13081543 34452408 PMC8402896

[6] Alt V, Gessner A, Merabishvili M, Hitzenbichler F, Mannala GK, Peterhoff D, Walter N, Pirnay J-P, Hiergeist A, Rupp M. 2024. Case report: local bacteriophage therapy for fracture-related infection with polymicrobial multi-resistant bacteria: hydrogel application and postoperative phage analysis through metagenomic sequencing. Front Med 11:1428432. doi:10.3389/fmed.2024.1428432 PMC11272550 39071087

[7] Ferry T, Bouar ML, Briot T, Roussel-Gaillard T, Perpoint T, Roux S, Ader F, Valour F, Kassai B, Boussaha I, et al.. 2024. Access to phage therapy at Hospices Civils de Lyon in 2022: implementation of the PHAGEinLYON clinic programme. Int J Antimicrob Agents 64:107372. doi:10.1016/j.ijantimicag.2024.107372 39491599

[8] Suh GA, Ferry T, Abdel MP. 2023. Phage therapy as a novel therapeutic for the treatment of bone and joint infections. Clin Infect Dis 77:S407–S415. doi:10.1093/cid/ciad533 37932115

[9] Young J, Lee SW, Shariyate MJ, Cronin A, Wixted JJ, Nazarian A, Rowley CF, Rodriguez EK. 2024. Bacteriophage therapy and current delivery strategies for orthopedic infections: a SCOPING review. J Infect 88:106125. doi:10.1016/j.jinf.2024.106125 38373574

[10] Pirnay J-P, Djebara S, Steurs G, Griselain J, Cochez C, De Soir S, Glonti T, Spiessens A, Vanden Berghe E, Green S, et al.. 2024. Personalized bacteriophage therapy outcomes for 100 consecutive cases: a multicentre, multinational, retrospective observational study. Nat Microbiol 9:1434–1453. doi:10.1038/s41564-024-01705-x 38834776 PMC11153159

[11] Aslam S, Roach D, Nikolich MP, Biswas B, Schooley RT, Lilly-Bishop KA, Rice GK, Cer RZ, Hamilton T, Henry M, et al.. 2024. *Pseudomonas aeruginosa* ventricular assist device infections: findings from ineffective phage therapies in five cases. Antimicrob Agents Chemother 68:e0172823. doi:10.1128/aac.01728-23 38470133 PMC10989018

[12] Chan BK, Turner PE, Kim S, Mojibian HR, Elefteriades JA, Narayan D. 2018. Phage treatment of an aortic graft infected with *Pseudomonas aeruginosa* Evol Med Public Health 2018:60–66. doi:10.1093/emph/eoy005 29588855 PMC5842392

[13] Jault P, Leclerc T, Jennes S, Pirnay J-P, Que Y-A, Resch G, Rousseau AF, Ravat F, Carsin H, Le Floch R, et al.. 2019. Efficacy and tolerability of a cocktail of bacteriophages to treat burn wounds infected by *Pseudomonas aeruginosa* (PhagoBurn): a randomised, controlled, double-blind phase 1/2 trial. Lancet Infect Dis 19:35–45. doi:10.1016/S1473-3099(18)30482-1 30292481

[14] Schooley RT, Biswas B, Gill JJ, Hernandez-Morales A, Lancaster J, Lessor L, Barr JJ, Reed SL, Rohwer F, Benler S, et al.. 2017. Development and use of personalized bacteriophage-based therapeutic cocktails to treat a patient with a disseminated resistant *Acinetobacter baumannii* infection. Antimicrob Agents Chemother 61:e00954-17. doi:10.1128/AAC.00954-17 28807909 PMC5610518

[15] Suh GA, Lodise TP, Tamma PD, Knisely JM, Alexander J, Aslam S, Barton KD, Bizzell E, Totten KMC, Campbell JL, et al.. 2022. Considerations for the use of phage therapy in clinical practice. Antimicrob Agents Chemother 66:e0207121. doi:10.1128/AAC.02071-21 35041506 PMC8923208

[16] Tkhilaishvili T, Winkler T, Müller M, Perka C, Trampuz A. 2019. Bacteriophages as adjuvant to antibiotics for the treatment of periprosthetic joint infection caused by multidrug-resistant *Pseudomonas aeruginosa*. Antimicrob Agents Chemother 64:e00924-19. doi:10.1128/AAC.00924-19 31527029 PMC7187616

[17] Schoeffel J, Wang EW, Gill D, Frackler J, Horne B, Manson T, Doub JB. 2022. Successful use of salvage bacteriophage therapy for a recalcitrant MRSA knee and hip prosthetic joint infection. Pharmaceuticals (Basel) 15:177. doi:10.3390/ph15020177 35215290 PMC8877365

